# Publication Trends for Alzheimer's Disease Worldwide and in China: A 30-Year Bibliometric Analysis

**DOI:** 10.3389/fnhum.2019.00259

**Published:** 2019-08-09

**Authors:** Rui Dong, Hong Wang, Jishi Ye, Mingshan Wang, Yanlin Bi

**Affiliations:** ^1^Department of Anesthesiology, Qingdao Municipal Hospital, Qingdao University, Qingdao, China; ^2^Department of Pediatrics, Qingdao Women and Children Hospital, Qingdao University, Qingdao, China; ^3^Department of Anesthesiology, Renmin Hospital of Wuhan University, Wuhan, China

**Keywords:** bibliometric analysis, China, PubMed, web of science, Alzheimer's disease, traditional Chinese medicine

## Abstract

**Background:** Alzheimer's disease, the most common form of dementia, has tremendous social and economic impact worldwide. This study aimed to analyze global trends in Alzheimer's disease research and to investigate China's contribution to this research.

**Methods:** The quantity and influence of publications related to Alzheimer's disease in China and elsewhere were compared. The Web of Science (WOS) and PubMed databases were searched from 1988 to 2017 using the terms “Alzheimer's disease” or “Alzheimers disease.” Global Alzheimer's disease publications were classified and analyzed. Keywords, countries, and institutions publishing articles on Alzheimer's disease were analyzed, and citations of these articles were examined.

**Results:** A total of 181,116 articles regarding Alzheimer's disease research were identified and analyzed. Neuroscience and neurology were the main research categories both globally and in China. Basic research dominated Alzheimer's publications, accounting for 30.93% of global publications and 95.31% of publications in China. A total of 8,935 journals published articles related to Alzheimer's disease. The journal *Neurobiology of Aging* published the most Alzheimer's disease-related articles, numbering 5,206 over the time period examined. The National Institutes of Health, the National Institute on Aging, and the Department of Health and Human Services jointly sponsored 11,809 articles, ranking first in the world. The National Natural Science Foundation of China funded the largest number of studies on Alzheimer's disease in China and recognized the importance of traditional Chinese medicine in Alzheimer's disease research.

**Conclusions:** The present study provides data for global researchers to understand research perspectives and develop future research directions. In recent years, Chinese researchers have contributed significantly to global Alzheimer's research. Still, strengthening international cooperation could improve the quality and number of publications regarding Alzheimer's disease.

## Introduction

Alzheimer's disease (AD) isa neuropathological disease involving progressive neurodegeneration. It initially impacts memory and leads to progressive and irreversible cognitive decline and functional impairment. In 2010, an estimated 503,400 deaths of Americans aged 75 years and older were attributed to AD (James et al., 2014). In Europe, a meta-analysis estimated the prevalence and incidence of AD in Europe to be 5.05% and 11.08 per 1,000 person-years, respectively (Niu et al., 2017). With increasing average life expectancy, AD is predicted to become a major socioeconomic burden in the near future and is projected to affect 131.5 million people worldwide by 2050 (Khan et al., 2017). Despite advancements in understanding AD, its exact etiology and pathogenesis remain unclear. Over the past three decades, our understanding of the mechanism of AD has increased and methods for the diagnosis and treatment of AD have been developed. Simultaneously, scientific and technological strength has developed in China. However, global and China-specific trends in AD research have not yet been described.

Bibliometrics, an emerging field of information science, is useful for gaining insights into the intellectual structure of an academic field. Bibliometric studies can identify hotspots in a specific research area. In addition, bibliometric analyses are often used to identify academically significant articles and landmark publications. Bibliometric analyses have been previously used to investigate the research output of various fields at the global, regional, and national levels, including neoplasms (Iqbal et al., 2019), parasite (Goarant et al., 2019), pituitary adenoma (Guo et al., 2018), and glioma (Burak Atci et al., 2019). However, a bibliometric study has not yet been conducted to understand the landscape of AD.

To evaluate the quantity and influence of global AD research, a bibliometric analysis was performed over the time period of 1988 to 2017. The present state and progress of AD research was examined both globally and in China.

## Materials and Methods

### Data Sources

Searches were performed on November 26, 2018, using the Web of Science (WOS) and PubMed databases. China-related data were derived from the latest National Natural Science Foundation funding results query system (http://www.letpub.com.cn/index.php?-page=grant). This study was not reviewed by an ethics committee as it did not involve any human subjects and all data are from public databases.

### Search Strategy

A pilot search in the WOS database indicated that the search term “Alzheimer's disease” does not retrieve all publications concerning Alzheimer's disease. Consequently, the topic (Alzheimer's disease or Alzheimers disease) was searched in the WOS database and results from 1988 to 2017 were compiled. This search included articles, reviews, proceedings, meeting abstracts, and letters. The PubMed database was searched for (Mesh = Alzheimer's disease) with publication dates (1988/01/01–2017/12/31). The document types retrieved included clinical trials, randomized controlled trials, basic research studies, and case reports. In order to screen for basic research, the species was identified as “other animals.” Two researchers independently screened, sorted, and extracted the data, with a third researcher participating in discussions to reach consensus when necessary. Bibliometric indicators, including total number of publications, citation frequency, citation frequency per article, h-index, research type, research orientation, research organization, journal, and funding support were extracted from the original data to quantitatively and qualitatively analyze the publications.

### Maps Based on Bibliographic Data

The title, authors, countries, institutions, abstract, and keywords of each article were extracted from the WOS database and analyzed using VOSviewer (v.1.6.11). For 1988, 1997, 2007, and 2017, 12 term maps were produced to illustrate networks of keywords, countries, and institutions, showing their co-occurrence and relative citation impacts. The methods and algorithms of VOSviewer have been described in detail in previous studies (van Eck and Waltman, 2017). Aside from the keywords map for 1988, keywords maps included keywords that occurred at least 30 times under binary counting. For institutions maps, all research institutions were mapped for 1988 and the top 1,000 research institutions in terms of total number of publications were mapped for 1997, 2007, and 2017. Countries maps included all countries involved in AD research.

### Statistical Analysis

This study mainly used descriptive statistical analysis. Trends over the three decades were analyzed using linear regression. Statistical significance was set at *P* < 0.05. SPSS statistical software (version 21.0, IBM, New York, USA) was used for data analysis.

## Results

### AD-Related Articles and Growth Trends

**Figure 1:**
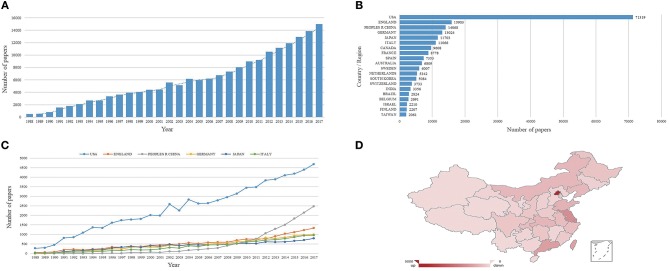
Alzheimer's disease (AD)-related articles globally and in China. **(A)** Global number of AD-related publications. Blue bars indicate the quantity of AD articles. **(B)** Sum of AD-related articles from the top 20 countries and territories. **(C)** AD articles published from the top six countries over time. **(D)** Heat map showing the distribution of AD publications in China.

This study identified a total of 181,116 articles related to AD published between 1988 and 2017. The number of publications regarding AD increased over time (*R*^2^ = 0.949, *P* < 0.001), from 481 in 1988 to 14,976 in 2017 (Figure 1A). A total of 180 countries or territories participated in AD research. By country, the most articles were published from the United States (71,319, 39.378%), followed by England (15,903, 8.781%), China (14,068, 7.767%), Germany (13,024, 7.191%), Japan (11,703, 6.462%), and Italy (11,066, 6.110%) (Figure 1B). As Figure 1C shows, publications regarding AD from individual countries also increased over time: United States (*R*^2^ = 0.986, *P* < 0.001), England (*R*^2^ = 0.929, *P* < 0.001), China (*R*^2^ = 0.679, *P* < 0.001), Germany (*R*^2^ = 0.976, *P* < 0.001), Japan (*R*^2^ = 0.952, *P* < 0.001), and Italy (*R*^2^ = 0.949, *P* < 0.001).

Strikingly, the number of AD-related publications from China increased after 2008 (*R*^2^ = 0.972, *P* < 0.001) (Figure 1C). Among Chinese provinces and municipalities, Beijing published the largest number of AD articles (Figure 1D).

### Citation and h-Index Analyses

**Figure 2:**
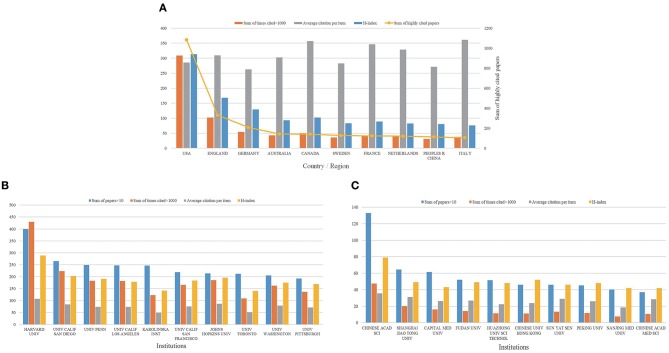
Citation and h-index analyses globally and in China. **(A)** Total citations, average citations per article, and h-index for AD articles from the top 10 countries. **(B)** Total citations, average citations per article, and h-index for AD articles from the top 10 global institutions by publication volume. **(C)** Total citations, average citations per articles, and h-index for AD articles from the top 10 Chinese institutions by publication volume.

The WOS database contained 1,712 highly cited articles related to AD. Figure 2A presents the 10 countries and territories publishing the most highly cited articles. The United States had 308,844 total citations and averaged 285.44 citations per item. The h-index for the United States was 313, higher than that for any other country or territory. Although China was ranked third by the number of articles published, the total citations and h-index of articles published in China (30,663 total citations, 271.35 citations per item and h-index of 80) were lower than those observed for other developed countries.

Literature on AD was published by about 49,836 different institutions from 1988 to 2017. Figure 2B presents the 10 institutions contributing the most research globally over this time frame. Eight of these 10 institutions are in the United States and the other two are in Canada (University of Toronto) and Sweden (Karolinska Institute). Harvard University was the top contributor to AD research with 3,998 publications, 429,976 total citations, and an h-index of 289, followed by the University of California at San Diego (2,654 articles, 223,686 total citations, h-index of 203), the University of Pennsylvania (2,491 articles, 183,107 total citations, h-index of 191), and the University of California, Los Angeles (2,475 articles, 182,475 total citations, h-index of 179). The publications identified 5,607 scientific research institutions in China involved in AD-related research. Figure 2C details the 10 Chinese institutions contributing the most publications to the field. The Chinese Academy of Sciences was the top Chinese contributor to AD research with 1,330 publications, 47,469 total citations, and an h-index of 79, followed by the Shanghai Jiao Tong University (643 articles, 20,080 total citations, h-index of 49), Capital Medical University (613 articles, 16,090 total citations, h-index of 43), and Fudan University (522 articles, 14,022 total citations, h-index of 49). While Chinese institutions are contributing meaningful research to the AD field, the top Chinese institutions are not yet contributing to AD literature at the level of the top global institutions.

### Study Category and Article Type Analyses

**Figure 3:**
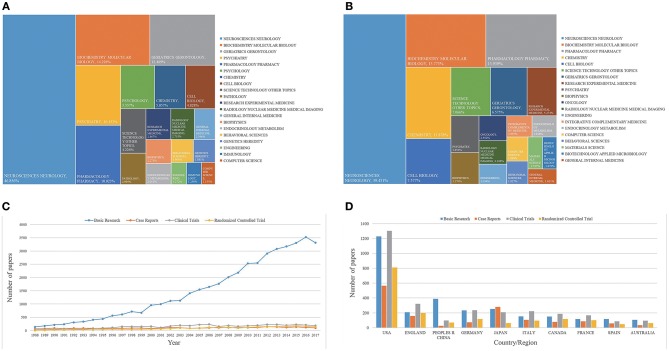
Research categories and article types globally and in China. **(A)** Research categories and fractions (percentages of research in each category) in global AD research. **(B)** Research categories and fractions (percentages of research in each category) in Chinese AD research. **(C)** Randomized controlled trials, clinical trials, case reports, and basic research worldwide from 1988 to 2017. **(D)** Article type analysis from the top 10 countries by publication volume from 1988 to 2017.

Globally, AD articles were categorized into 144 study types. As shown in Figure 3A, the most frequently observed categories were neuroscience/neurology with 84,864 articles (46.856%), followed by biochemistry/molecular biology with 25,769 articles (14.228%), geriatrics/gerontology with 22,620 articles (12.489%), psychiatry with 18,389 articles (10.153%), and pharmacology/pharmacy with 18,154 articles (10.023%). As shown in Figure 3B, the first several research categories of Chinese AD literature do not differ from the top several categories globally. The top five research categories in China were neuroscience/neurology with 5,550 articles (39.451%), biochemistry/molecular biology with 2,219 articles (15.773%), pharmacology/pharmacy with 1,961 articles (13.939%), chemistry with 1,671 articles (11.878%), and cell biology with 1,066 articles (7.577%).

In the past 30 years, according to the PubMed database search, basic research accounted for 39,429 articles (30.93%), and the proportion of this literature increased over time (*R*^2^ = 0.949, *P* < 0.001), as shown in Figure 3C. Additionally, 2,786 case reports (2.19%), 4,077 clinical trials (3.20%), and 2,242 randomized controlled trials (1.76%) were published in the AD field. In China, 3,884 basic research articles were published between 1988 and 2017, accounting for 95.31% of China's AD articles. The number of publications of each article type from the United States far exceeded that of any other country or territory, as shown in Figure 3D. China has become the country with the second largest total number of published AD articles; however, Chinese articles contain much more basic research and less clinical research compared to global publications.

### Keywords, Country, and Institution Map Analyses

**Figure 4:**
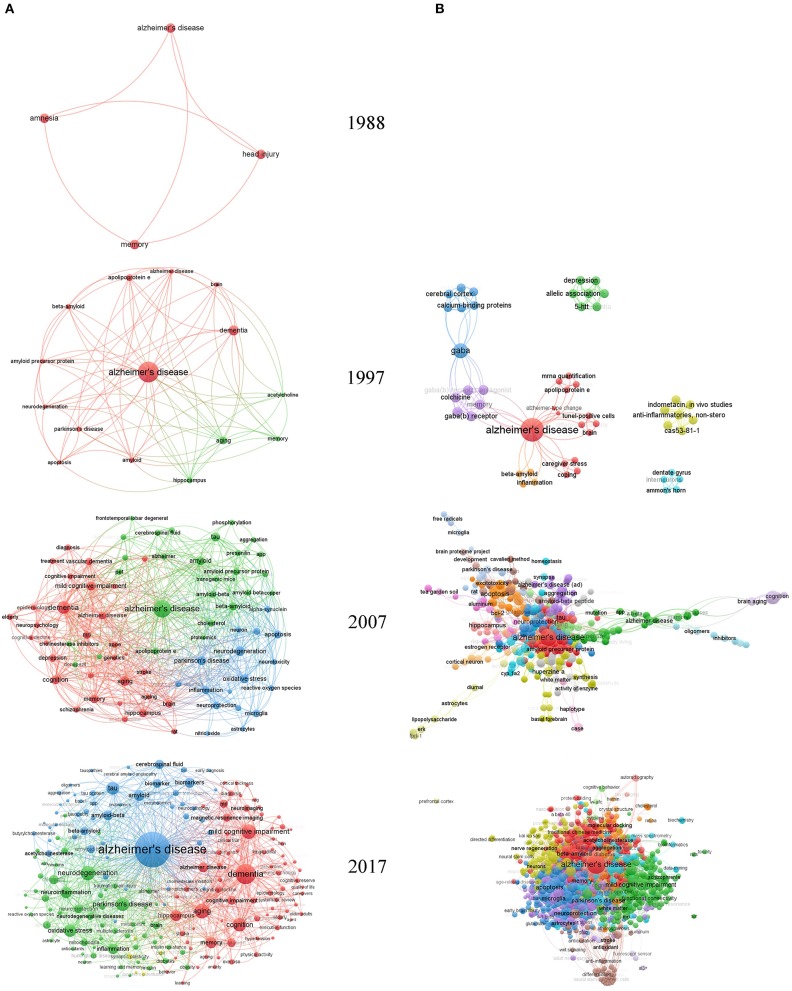
Keyword maps globally and in China. **(A)** Global keyword maps. **(B)** Keyword maps in China. In this visualized network, each keyword is represented by a node. Node size reflects the number of publications in the node, and the distance between two nodes indicates the connectivity of the nodes as determined by co-occurrences. The larger the number of publications for which two nodes are both found, the stronger the relationship between the nodes. VOSviewer has its own clustering technique based on citation relations between clusters. Colors represent groups of nodes that are relatively strongly related to each other.

Global keywords maps illustrating the co-occurrence network of keywords for 1988, 1997, 2007, and 2017 are shown in Figure 4A. In 1988, AD-related research included only four keywords: Alzheimer's disease, amnesia, memory, and head injury. By 1997, the number of keywords had climbed to 4,137. AD-related research had expanded to the protein level and included topics such as acetylcholine, amyloid, apoptosis, amyloid precursor protein, beta-amyloid, and apolipoprotein E. Additionally, relationships between AD and neurodegeneration, Parkinson's disease, the hippocampus, and aging had been reported by 1997. By 2007, the number of keywords had increased to 10,713, including some of the same keywords from previous decades such as dementia, neurodegeneration, aging, hippocampus, aging, amyloid, apoptosis, and beta-amyloid. In addition, a series of new keywords including oxidative stress, mild cognitive impairment, tau, inflammation, microglia, neuroprotection, mitochondria, MRI, schizophrenia, cholesterol, and diabetes had appeared, and new pathophysiologic mechanisms of AD had been recognized. By 2017, the number of keywords had reached 22,641. New keywords had emerged such as acetylcholinesterase, alpha-synuclein, amyotrophic lateral sclerosis, functional connectivity, blood–brain barrier, frontotemporal dementia, neuroimaging, positron emission tomography, and biomarkers, reflecting the enriched content of AD research. New pathophysiologic mechanisms have been recognized and functional neuroimaging techniques have been gradually introduced to evaluate AD.

In 1990, Chinese scholars described their AD research investigating the prevalence of dementia and AD in Shanghai in *Annals of Neurology* (Zhang et al., 1990). Therefore, we analyzed the keyword and institution maps for 1997, 2007, and 2017 in China, as shown in Figure 4B. In 1997, AD research in China covered 51 keywords, such as GABA, colchicine, 5-HTT, allelic association, beta-amyloid, inflammation, caregiver-stress, and dentate gyrus. By 2007, AD-related research in China was associated with 901 keywords, including apoptosis, tau, beta-amyloid, amyloid precursor protein, neuroprotection, oxidative stress, aggregation, hippocampus, and tea garden soil. By 2017, the number of keywords in China had reached 5,079, reflecting new keywords such as neurodegeneration, mild cognitive impairment, nerve regeneration, meta-analysis, white matter, traditional Chinese medicine (TCM), Kai Xin San, molecular docking, and functional connectivity. The relationship between TCM and AD is characteristic of Chinese research.

**Figure 5:**
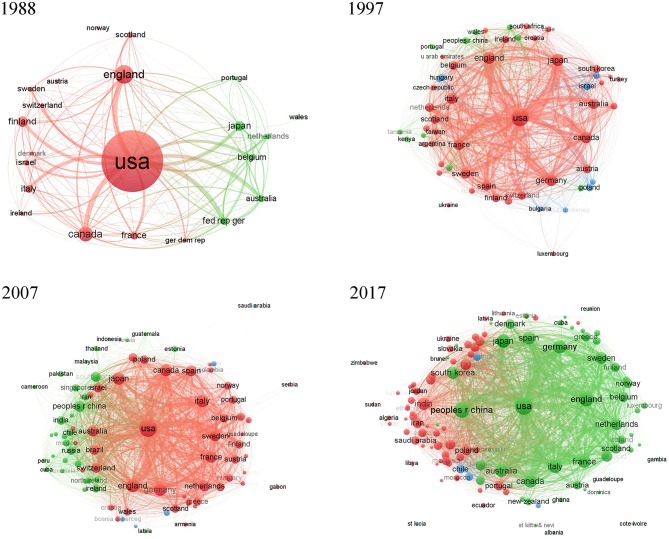
Global countries and regions maps. In this visualized network, each country or region is represented by a node. Node size reflects the number of publications in the node, and two nodes are connected by a curved line if either of them cites the other. Curved lines between nodes reflect the connectivity of nodes, with line thickness representing the number of citations between two nodes. VOSviewer has its own clustering technique based on citation relations between clusters. Colors represent groups of nodes that are relatively strongly related to each other.

**Figure 6:**
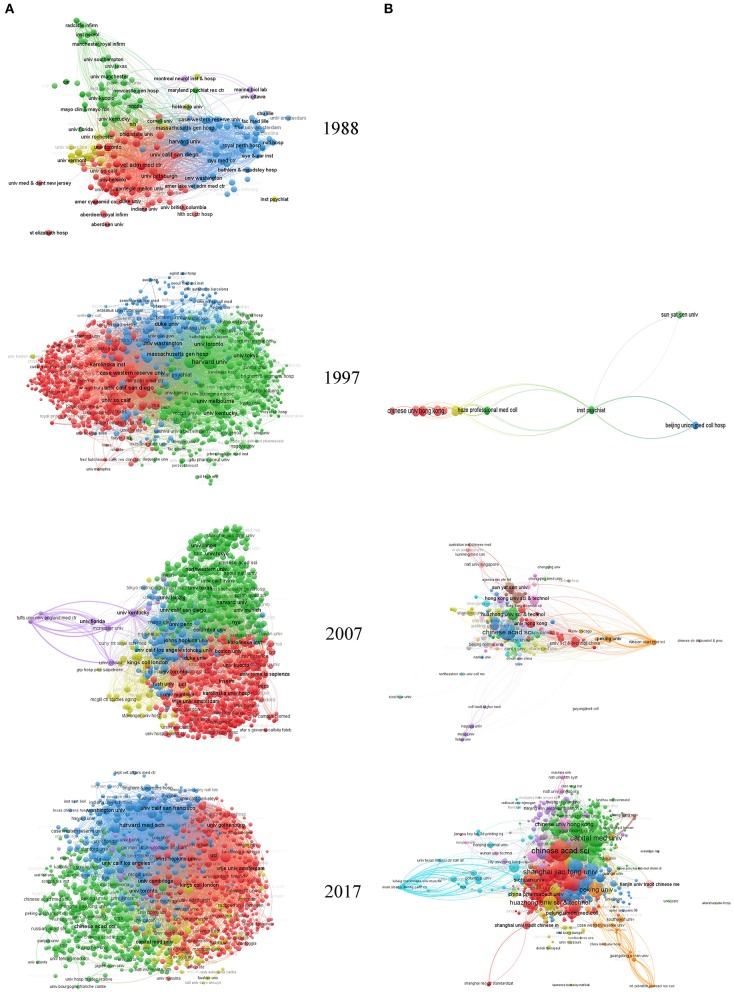
Institution maps globally and in China. In this visualized network, each institution is represented by a node. Node size reflects the number of publications in the node, and two nodes are connected by a curved line if either of them cites the other. Curved lines between nodes reflect the connectivity of nodes, with line thickness representing the number of citations between two nodes. VOSviewer has its own clustering technique based on citation relations between clusters. Colors represent groups of nodes that are relatively strongly related to each other. **(A)** Global institution maps. **(B)** Institution maps in China.

Figure 5 shows the bibliographic network of countries and regions in AD research in 1988, 1997, 2007, and 2017. As seen in this visualization, the United States, England, Canada, Japan, Italy, France, Australia, Germany, and Switzerland each developed more AD research over time and also increased research collaborations among countries. Globally, most institutions contributing to AD research are located in the European Union and the United States (Figure 6A). By 2017, the number of AD publications from China had increased significantly, and Chinese research institutes with a focus on AD research had been established (Figures 6A,B).

### Journal and Research Funding Agency Analyses

**Figure 7:**
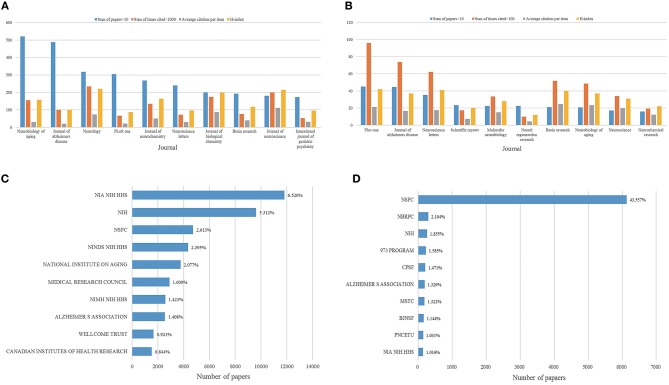
Highly contributing journals and funding systems to AD research publications globally and in China. **(A)** Sum of publications, total citations, average citations per article, and h-index for AD research journals worldwide. **(B)** Sum of publications, total citations, average citations per article, and h-index for AD research journals in China. **(C)** Top 10 funding systems contributing to global AD research. **(D)** Top 10 funding systems contributing to AD research in China. NIA, National Institute on Aging; NIH, National Institutes of Health; NSFC, National Natural Science Foundation of China; NINDS, National Institute of Neurological Disease and Stroke; HHS, Department of Health and Human Services; NIMH, National Institute of Mental Health; NBRPC, National Basic Research Program of China; 973 PROGRA, National High Technology Research and Development Program of China; CPSF, China Postdoctoral Science Foundation; MSTC, Ministry of Science and Technology of China; BJSF, Beijing Natural Science Foundation; PNCETU, Program for New Century Excellent Talents in University.

A total of 8,935 journals published AD-related articles, and 1,966 journals published articles by Chinese researchers. The 10 journals publishing the largest number of reports are presented in Figure 7A. *Neurobiology of Aging* was the most active journal on AD research with 5,206 articles (2.874%), followed by the *Journal of Alzheimer's Disease* with 4,873 articles (2.691%), *Neurology* with 3,179 articles (1.755%), *PLoS ONE* with 3,044 articles (1.681%), and *Journal of Neurochemistry* with 2,678 articles (1.479%). The journals publishing the most AD research from China were *PLoS ONE* with 450 articles (3.199%), the *Journal of Alzheimer's Disease* with 447 articles (3.177%), *Neuroscience Letters* with 354 articles (2.516%), *Scientific Reports* with 232 articles (1.649%), and *Molecular Neurobiology* with 224 articles (1.592%), as shown in Figure 7B. Globally, the National Institutes of Health, the National Institute on Aging, and the Department of Health and Human Services jointly sponsored 11,809 articles, ranking first in the world for funding published AD research, as shown in Figure 7C. In China, the National Natural Science Foundation of China (NSFC) funded the most research, producing 6,128 articles (43.557%), as shown in Figure 7D.

**Figure 8:**
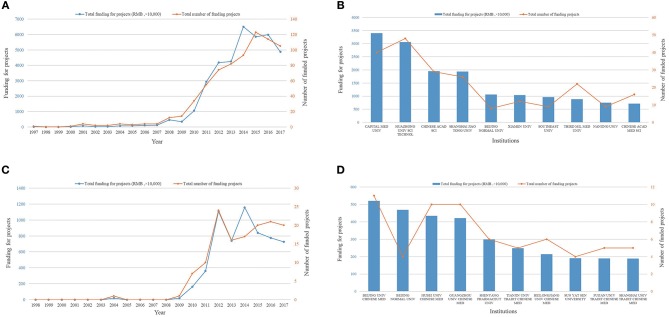
NSFC funding, NSFC-funded institutions, and NSFC growth trends in China. **(A)** NSFC funding from 1997 to 2017. The blue curve indicates the total amount of funding and the orange curve indicates the number of projects. **(B)** NSFC funding of AD research at different Chinese institutions. Blue bars indicate the total amount of funding and the orange curve indicates the number of projects. **(C)** NSFC funding for studies on the mechanism of traditional Chinese medicine in AD from 1997 to 2017. The blue curve indicates the total amount of funding and the orange curve indicates the number of projects. **(D)** NSFC funding for studies on the mechanism of traditional Chinese medicine in AD at different Chinese institutions. Blue bars indicate the total amount of funding and the orange curve indicates the number of projects.

Total NSFC funding and the number of AD research projects funded in China have grown since 2008 (*R*^2^ = 0.914, *P* < 0.001; *R*^2^ = 0.804, *P* < 0.001). These correlational analysis results are summarized in Figure 8A. Among all Chinese research institutions, Capital Medical University ranked first for research funding with 40 projects, totalling 33.97 million yuan, as shown in Figure 8B. In addition, funding for the study of TCM in the AD field has increased substantially since 2008, as shown in Figure 8C. Beijing University of Traditional Chinese Medicine ranked first in AD research using TCM, with 11 projects and a total funded amount of 5.2 million yuan, as shown in Figure 8D. These results indicate that TCM receives considerable attention from the Chinese government and appreciable financial support from the NSFC.

### Most-Cited AD Articles Analysis

Tables 1, 2 present details of the 10 most-cited articles on AD, globally and in China. The 10 most-cited articles globally had 4,938–13,478 citations. Four of the world's most-cited articles are reviews, and three of China's most-cited articles are reviews. China's most-cited articles were primarily published after 2010. A majority of the most-cited articles from China were products of international collaboration, with only one of the Chinese most-cited articles independently completed by Chinese scholars.

**Table 1:** The top 10 most frequently cited papers on AD in the world.

**Title**	**Authors**	**Journal**	**Year**	**Type**	**Times cited**
Initial sequencing and analysis of the human genome	Lander, E. S. et al.	Nature	2001	Review	13,478
Neuropathological staging of Alzheimer-related changes	Braak, H. et al.	Acta Neuropathologica	1991	Review	7,979
Medicine–the amyloid hypothesis of Alzheimer's disease: progress and problems on the road to therapeutics	Hardy, J. et al.	Science	2002	Review	7,847
Free radicals and antioxidants in normal physiological functions and human disease	Valko, M. et al.	The International Journal of Biochemistry and Cell Biology	2007	Review	6,005
Accuracy of clinical diagnosis of idiopathic Parkinson's disease: a clinico-pathological study of 100 cases.	Hughes, A. J. et al.	Journal of Neurology, Neurosurgery and Psychiatry	1992	Article	5,678
Gene dose of apolipoprotein E type 4 allele and the risk of Alzheimer's disease in late onset families.	Corder, E. H. et al.	Science	1993	Article	5,677
Global and regional mortality from 235 causes of death for 20 age groups in 1990 and 2010: a systematic analysis for the Global Burden of Disease Study 2010	Lozano, R. et al.	Lancet	2012	Article	5,142
Mild cognitive impairment - clinical characterization and outcome	Petersen, R. C. et al.	Archives of Neurology	1990	Article	4,929
Mutation in the alpha-synuclein gene identified in families with Parkinson's disease	Polymeropoulos, M. H. et al.	Science	1997	Article	4,899
The Montreal Cognitive Assessment, MoCA: a brief screening tool for mild cognitive impairment	Nasreddine, Z. S. et al.	Journal of the American Geriatrics Society	2005	Article	4,398

**Table 2:** The top 10 most frequently cited papers on AD China.

**Title**	**Authors**	**Journal**	**Year**	**Type**	**Times cited**
Initial sequencing and analysis of the human genome	Lander, E. S. et al.	Nature	2001	Review	13,478
Global and regional mortality from 235 causes of death for 20 age groups in 1990 and 2010: a systematic analysis for the Global Burden of Disease Study 2010	Lozano, R. et al.	Lancet	2012	Article	5,142
Global prevalence of dementia: a Delphi consensus study	Ferri, C. P. et al.	Lancet	2005	Article	2,925
Global, regional, and national age-sex specific all-cause and cause-specific mortality for 240 causes of death, 1990-2013: a systematic analysis for the Global Burden of Disease Study 2013	Naghavi, M. et al.	Lancet	2015	Article	2,558
Glutathione metabolism and its implications for health	Wu, G. Y. et al.	Journal of Nutrition	2004	Article	1,593
Integrative analysis of 111 reference human epigenomes	Kundaje, A. et al.	Nature	2015	Article	1,185
Global, regional, and national life expectancy, all-cause mortality, and cause-specific mortality for 249 causes of death, 1980–2015: a systematic analysis for the Global Burden of Disease Study 2015	Wang, H. D. et al.	Lancet	2016	Article	1,054
REST: a toolkit for resting-state functional magnetic resonance imaging data processing	Song, X. W. et al.	PLoS ONE	2011	Article	995
Global, regional, and national incidence, prevalence, and years lived with disability for 310 diseases and injuries, 1990–2015: a systematic analysis for the Global Burden of Disease Study 2015	Vos, T. et al.	Lancet	2016	Article	879
Neuroinflammation in Alzheimer's disease	Heneka, M. T. et al.	Lancet Neurology	2015	Review	851

## Discussion

The present study provides a comprehensive overview of the global development of AD research and summarizes the contribution of China to AD research over the last three decades. In the past 30 years, the global volume of AD research has increased tremendously. A linear growth was observed in the number of AD publications over the survey period, consistent with publications in other neuroscience research fields (Yeung et al., 2017a). Most research originated in the United States, highlighting that country's important role in AD research. Our findings suggest that healthcare expenditures are substantially related to the economic power of a country. With its rapid growth in science and technology, China has made great strides and now holds a respectable position in AD research among developing countries, but a gap still exists between China and developed countries in terms of the academic level of AD research. In recent years, especially since 2008, the Chinese government has supported the development of AD research: the number of projects funded by NSFC has increased, and the amount of funding from the NSFC has expanded. At the same time, the lens of TCM has been used to investigate the pathological mechanisms and prevention of AD.

Global AD research has developed appreciably, with 481 articles published in 1988 and 14,985 in 2017. Based on results of these studies, the pathological mechanisms of AD are becoming more and more defined, including the accumulation of pathological misfolded tau (Leuzy et al., 2019), the amyloid-β cascade hypothesis (Panza et al., 2019), circadian dysrhythmia (Van Erum et al., 2018), gut microbiota (Jiang et al., 2017), cholinergic neuron death and acetylcholine deficiency (Anand et al., 2012), and chronic inflammation and oxidative stress (Jaworski et al., 2011). In line with the mechanisms mentioned above, drugs to treat AD have been developed to reduce tau protein abnormalities, inhibit amyloid-β protein production, treat circadian rhythm disorder, modulate the structure and metabolism of gut microbiota, increase acetylcholine synthesis, protect cholinergic neurons, and promote anti-inflammatory and anti-oxidant compounds. At present, there is no cure for AD; treatments for AD only alleviate disease symptoms, and many treatments are effective in some AD patients for a limited time. Despite research efforts, no disease-modifying treatments are available and the underlying mechanisms of AD and optimal treatment targets have not been fully elucidated.

Early non-invasive examination tools such as transcranial sonography (Favaretto et al., 2018) have been improved, and functional neuroimaging techniques have been gradually applied in AD research (Dennis and Thompson, 2014; delEtoile and Adeli, 2017). The overarching goals of AD research are to determine when and how interventions can slow or stop cognitive decline in patients, and to identify early signs of decline and treatment response. Phase II results of new treatments and several therapies in phase III clinical trials are encouraging. Previous studies revealed that pathophysiological changes of AD take place decades before clinical symptoms of dementia appear (Morris, 2005). This extended preclinical phase of AD provides a critical opportunity for disease-modifying agents to halt or slow the progression of AD (Sperling et al., 2011). We therefore optimistically anticipate more effective therapies in the foreseeable future. Partnerships between pharmacy, biotechnology, physics, chemistry, electronic informatics, botany, and medicine are growing. These will enable treatments with a range of targets to be developed, with an emphasis on drug repositioning and novel drug discovery. More investment is urgently needed to enable more treatments to be studied in clinical trials. Some form of preventative treatment strategy, possibly specialized combination therapies with multiple targets, may be available in the next decade.

Research results must be published in the form of journal articles. Globally, 8,935 journals have published AD-related articles, with the 10 journals publishing the largest number of articles in this field contributing only 15.925% of published AD research. Journals publishing large numbers of AD-related articles, such as *Neurobiology of Aging, Journal of Alzheimer's Disease, Neurology, PLoS ONE, Journal of Neurochemistry*, and *Neuroscience Letters*, allow different viewpoints to be freely exchanged. The journals publishing the 10 most-cited articles and those publishing the largest number of articles are not the same, for complex reasons. The journals publishing the 10 most-cited articles have long histories, such as the *Lancet*, published since 1823, *Nature*, published since 1869, *Science*, published since 1880, and the *Journal of Neurology, Neurosurgery, and Psychiatry*, published since 1920. Being published in such well-established journals might have allowed these articles to accumulate a large number of citations, consistent with suggestions from a previous neuroscience study (Yeung et al., 2017b). By contrast, citations can have a snowball effect, since articles with higher impact factors are likely to be cited more often (Lefaivre et al., 2011; Yeung et al., 2017b). These highly cited articles should be familiar to every AD researcher and are critical for understanding the key messages in this field.

Large clusters in keyword co-occurrence networks indicate important research hotspots. Worldwide, the number of keywords in AD-related literature has grown from 4 in 1988 to 22,641 in 2017, illustrating the enriched content of this research. Popular research topics include beta-amyloid, tau, aging, the hippocampus, neurodegeneration, apoptosis, neuroprotection, neuroinflammation, and neurodegenerative diseases. These represent good choices for strong AD research teams but may be risky for less established research teams owing to the difficulty of securing funding and making research breakthroughs in these relatively underdeveloped areas. Less developed research areas include neuroimaging, functional connectivity, iron, reactive oxygen species, alpha-synuclein, proteostasis, phytochemicals, homocysteine, voxel-based morphometry, angiogenesis, and sleep. Dynamic research trends can be observed to understand changes in the clustering network and to make informed decisions on research directions. Regardless, each of these areas helps to improve our understanding of the pathophysiological mechanisms of AD.

Despite changes in research topics, the countries and institutes producing the most AD literature did not change significantly between 1988 and 2017. The United States, Canada, Japan, Australia, and European Union countries continue to be major contributors to AD research. Remarkably, these countries were previously identified as major contributors to neuroscience research (Yeung et al., 2017a), indicating that these countries and their institutes occupy core positions in global research. Prior to 2008, AD research in China was progressing slowly. Since 2008, Chinese AD research has grown substantially. A possible explanation for this is that the Chinese government established the Central Coordination Group for Talents Work and implemented the Recruitment Program of Global Experts in 2008. By 2018, the program has attracted about 6,000 Chinese scientists, entrepreneurs, and scholars living abroad to return to China. Another possible explanation for this growth is the increased financial aid to AD research and the enhanced funding system for AD research. Together, these results provide insights into the importance of both talent and financial support for developing scientific research. The economic power of a country has a direct bearing on its medical research expenditures (Zhang et al., 2017; Ye et al., 2018). In recent years, China's economic power has grown rapidly and the country has become the world's second-biggest economy. This could greatly boost AD research in China. The NSFC, with 18 sponsor systems covering all scientific subjects, is a bellwether of scientific research grants in China. In addition to support for local scientific researchers, the NSFC also provides substantial support to international collaborative projects and Chinese scientists returning from overseas studies. Beyond the China-wide funding system, the country has established a medical research funding system at the provincial, municipal, and district levels, with funds such as the Zhejiang Provincial Natural Science Foundation, and the Qingdao Municipal Source Innovation Project Fund. Thanks to these policies and funding systems, a large number of outstanding scholars have returned to China and have promoted AD research because of the concepts, techniques, international academic exchanges, and collaborations they bring with them. Additionally, the huge population base in China provides an unparalleled advantage for AD clinical research. According to data from the Sixth Census of China's National Bureau of Statistics (2010), 13.26% of China's population is over 60 years old; according to the Statistical Bulletin of Social Services Development published by the Ministry of Civil Affairs in 2016, 16.7% of China's population is over 60 years old. It estimated that, by 2030, the proportion of the total population over 60 years old will exceed 20% of the total population. So far, researchers in China have relatively low output in clinical trials and randomized controlled trials compared to basic research. This is largely due to the NSFC principally supporting basic scientific research. Clinical study of AD in China has great potential, and platforms for clinical cooperation, databases, and analysis systems should be planned to unite and integrate this massive clinical potential.

However, the gap between China and leading countries in AD research cannot be ignored. While the total number of AD articles from China has increased dramatically in recent years, the citation frequency and h-index remain low. In terms of research types, Chinese publications are highly focused on basic research, and in earlier years, Chinese AD research lagged behind global AD research. For example, in 1988, no AD-related research was published in China; in 1997, apoptosis and neurodegeneration appeared on global keywords co-occurrence maps, while these keywords only appeared on China's keywords co-occurrence maps in 2007. Studies associated with MRI and schizophrenia were conducted globally in 2007, but these keywords only appeared in China's keywords co-occurrence maps in 2017. Only one Chinese research institute has entered the top 80 global institutions contributing to AD research. In our bibliometric analysis, China's scientific research institutions impacted the world institutions map only in 2017. There are few AD-related international journals in China; creation of several international AD-related journals might attract submissions and encourage academic exchange. Finally, TCM research provides a unique opportunity for AD research in China. TCM has been prescribed in the Chinese community for more than 2000 years, and many herbal compounds are regarded as promising anti-AD drugs (Howes et al., 2017). However, unknown molecular targets and mechanisms, incorrect dosage, and high toxicity of herbal concoctions due to their complex formulations have hindered the therapeutic development of TCM. Chinese researchers could achieve breakthroughs in pharmacological research in TCM, eventually developing TCM as alternative medicines to modulate AD.

The bibliometric analysis had some limitations. First, bibliometric analyses cannot necessarily measure the validity of or the scientific quality of publications and instead must focus on the impact of the research. A highly cited publication may not necessarily be of high scientific quality. An article's citation count depends on a variety of factors, including journal type, research model, and self-citing rate. Second, delayed publication collections from the WOS and PubMed databases could also cause bias in the study. Third, the WOS and PubMed databases mainly included literature written in English, excluding many non-English publications. Fourth, the WOS and PubMed databases classify literature types differently, so the literature identified from different databases cannot be compared mechanically. Readers should note these confounding factors when interpreting the results from the present study.

## Conclusions

The current study provides a comprehensive perspective on AD research and illustrates that this is a well-developed and promising research field. The most frequent study category was neuroscience/neurology. *Neurobiology of Aging* was the journal publishing the most AD research. Research institutes and research funding agencies from North America and Europe constitute the core research forces, and the United States contributed the most publications. Although, China has gradually become a critical force in AD research, China still lacks high-level research institutions and literature. This bibliometric analysis can guide future research and serve as an educational guide for trainees.

## Data Availability

All datasets generated for this study are included in the manuscript and/or the supplementary files.

## Author Contributions

RD and HW participated in study design, data collection, statistical analysis, and manuscript preparation. JY performed data collection and statistical analysis. MW participated in data check. YB contributed significantly to the preparation of the study and its conception. All authors read and approved the final manuscript.

### Conflict of Interest Statement

The authors declare that the research was conducted in the absence of any commercial or financial relationships that could be construed as a potential conflict of interest.

## Abbreviations

AD: Alzheimer's disease
NIA: National Institute on Aging
NIH: National Institutes of Health
NSFC: National Natural Science Foundation of China
NINDS: National Institute of Neurological Disease and Stroke
HHS: (Department of) Health and Human Services
NIMH: National Institute of Mental Health
NBRPC: National Basic Research Program of China
973 PROGRA: National High Technology Research and Development Program of China
CPSF: China Postdoctoral Science Foundation
MSTC: Ministry of Science and Technology of China
BJSF: Beijing Natural Science Foundation
PNCETU: Program for New Century Excellent Talents in University.
